# Gut Microbial Signatures in Sporadic and Hereditary Colorectal Cancer

**DOI:** 10.3390/ijms22031312

**Published:** 2021-01-28

**Authors:** Giorgia Mori, Maria Rosalia Pasca

**Affiliations:** Department of Biology and Biotechnology “Lazzaro Spallanzani”, University of Pavia, 27100 Pavia, Italy

**Keywords:** gut microbiota, colorectal cancer, microbial biomarkers, Lynch syndrome, hereditary colorectal cancer

## Abstract

Colorectal cancer (CRC) is the fourth most common cause of cancer-related death and the third most common cancer in the world. Depending on the origin of the mutation, colorectal carcinomas are classified as sporadic or hereditary. Cancers derived from mutations appearing during life, affecting individual cells and their descendants, are called sporadic and account for almost 95% of the CRCs. Less than 5% of CRC cases result from constitutional mutations conferring a very high risk of developing cancer. Screening for hereditary-related cancers is offered to individuals at risk for hereditary CRC, who have either not undergone genetic evaluation or have uncertain genetic test results. In this review, we briefly summarize the main findings on the correlation between sporadic CRC and the gut microbiota, and we specifically focus on the few evidences about the role that gut microorganisms have on the development of CRC hereditary syndromes. The characterization of a gut microbiota associated with an increased risk of developing CRC could have a profound impact for prevention purposes. We also discuss the potential role of the gut microbiota as therapeutic treatment.

## 1. Introduction

In 2018, the International Agency for Research on Cancer and the World Health Organization GLOBOCAN database rated colorectal cancer (CRC) as the third most common cancer worldwide (1.8 million new cases), only preceded by lung (2.09 million new cases) and breast cancers (2.08 million new cases) [1,2]. Indeed, approximately 10% of all annually diagnosed cancers and cancer-related deaths belong to CRC worldwide [2].

Specifically, rates are substantially higher in males than in females. Furthermore, CRC is the third most common type of cancer in men (1.03 million new cases/year), after lung and prostate cancers, and the second most frequent malignancy in women (0.82 million new cases/year) after breast cancer [1,2,3,4]. In women, incidence and mortality are approximately 25% lower than in men [1,4]. The incidence of CRC is predicted to reach 2.5 million new cases in 2035 worldwide [1].

These rates also vary geographically, with the highest rates seen in the most developed countries such as Australia and New Zealand, Europe, and North America, and the lowest rates found in Africa and South-Central Asia [3]. These variations appear to be linked to differences in dietary and environmental factors which, together with hereditary risk factors, can increase the likelihood of developing CRC [4,5,6].

According to the International Agency for Research on Cancer, the risk of developing CRC is mainly influenced by environmental causes, although 5–10% of CRCs are caused by genetic factors, such as hereditary syndromes and familial history [1] (Table 1).

Although the majority of CRCs are sporadic, a low percentage of CRC patients are affected by a hereditary CRC syndrome [19]. Hereditary CRC syndromes that are inherited in an autosomal-dominant fashion can be divided in non-polyposis (e.g., Lynch syndrome (LS)) and polyposis syndromes and are linked to a high risk of developing CRC [7,20].

Among hereditary CRC syndromes, LS accounts for approximately 3% of all cases and is caused by a germline mutation in one allele of the DNA mismatch repair (MMR) genes, such as *MLH1*, *MSH2*, *MSH6*, or *PMS2*. Microsatellite instability (MSI) is a hypermutable phenotype linked to LS caused by the loss of DNA mismatch repair activity and it is also present in 15% of sporadic CRCs [6,8,21,22,23]. LS patients are also at an increased risk for endometrial cancer and other neoplasm formations (e.g., cancers of the small bowel, stomach, ovaries, brain, renal pelvis and ureter, hepatobiliary system, breast and prostate) [1,6,24].

The second most common hereditary syndrome is the familial adenomatous polyposis (FAP) and its variants (Gardner syndrome, Turcot syndrome, and attenuated FAP), which account for less than 1% of CRCs. FAP is caused by germline mutations in the adenomatous polyposis coli (*APC*) gene which is located on chromosome 5. In typical FAP, numerous colonic adenomas appear at an average age of approximately 16 years and CRC occurs by age 45. Attenuated FAP carries a high CRC risk and it is characterized by fewer adenomas and an older average age than FAP syndrome [25,26].

MUTYH-associated polyposis is an autosomal-recessive syndrome due to biallelic germline mutations in the base excision repair gene *MUTYH*. This syndrome presents a polyposis phenotype with fewer than 500 adenomas [26,27,28].

Recently, new adenomatous polyposis predisposing genes (*POLE* and *POLD1*, *MSH3*, and *NTHL*-associated polyposis) have been considered as part of next-generation panels under investigation [29].

Sporadic CRC accounts for the majority of CRC cases (about 95%) [1]. As already described, most sporadic CRCs develop through the so-called adenoma–carcinoma sequence, where polypoid adenomas progress into high-grade dysplastic adenoma, eventually leading to the formation of malignant forms [30]. This process is a slow multistep mechanism that involves specific mutations occurring at specific times [31]. Therefore, early CRC detection remains a priority for CRC control.

It is noteworthy that the development of sporadic CRCs can occur through three main genetic/epigenetic pathways, namely the CIN, MSI and CIMP pathways [1,2,3,4]. The chromosomal instability (CIN) pathway accounts for 65–75% of sporadic CRCs [32] and is characterized by an alteration in chromosome number or structure, loss of heterozygosity and aneuploidy, and mutations in the APC/β-catenin-dependent Wnt signaling pathway [33]. The CpG island methylator phenotype (CIMP) pathway accounts for 20–30% of sporadic CRC cases and it is associated with the serrated neoplasia CRC pathway [34,35], which includes sessile serrated polyps as precursors. Typically, sporadic CRCs arise from traditional adenomas in the adenoma-carcinoma sequence (CIN) and from sessile serrated polyps in the serrated neoplasia pathway (CIMP). In particular, an aberrant colonic crypt evolves into a neoplastic precursor lesion (a polyp or an adenoma), and eventual progresses to CRC over an estimated 10–15-year period. For the majority of CRCs, the cell of origin, residing in the base of the colonic crypt, is the CRC precursor and corresponds to the result of the progressive accumulation of genetic and epigenetic alterations that inactivate tumor-suppressor genes and activate oncogenes [6].

Finally, the microsatellite instability (MSI) pathway occurs in 10–15% of sporadic CRC cases, usually as a result of hypermethylation of the *MLH1* MMR gene. Sporadic MSI tumors are more common in the right colon and in female patients [6].

Among the environmental factors having a role in CRCs, the microorganisms living in our gut (gut microbiota) have recently been recognized as being potentially involved in CRC development [12,36]. In recent years, the human gut microbiota got a foothold in the list of the risk factors responsible for the onset and evolution of both forms of CRC (hereditary and sporadic) (Table 1).

The gut microbiota includes bacteria, archaea, phages, viruses and fungi, residing in our intestine. The microbiome refers to the microbiota’s genetic information [37].

The gut microbiota includes many microorganisms, comprising commensals and pathogens that interact closely with host intestinal cells, by contributing to physiology and pathology of the gut [12,38]. Among gut microorganisms, it is possible to find commensals that may cause a disease under certain conditions as well as some symbionts that could vary from mutualism and commensalism to parasitism [38]. The colorectum interacts with about 3 × 10^13^ bacteria that are important for several physiological functions, such as energy harvest and immune maturation, and the alteration of the bacterial relative abundance, and consequently of physiological function equilibrium, could contribute to cause intestinal and extraintestinal diseases [6].

The alteration of the homeostasis of the gut microbiota, also called dysbiosis, is considered a potential cause and signature of risk of disease [6]. To this purpose, Hippocrates had already stated that “all disease begins in the gut.” Recently, gut microbial dysbiosis has been linked to several clinical conditions including cardiovascular diseases, metabolic diseases, neuropsychiatric diseases, inflammatory bowel disease, *Clostridium difficile* infections, and some cancers in the colorectum, liver, biliary tract and breast [38,39,40,41,42,43,44].

Comprehensive metagenomic and metabolomic analyses might therefore provide a valid approach for the understanding of CRC development through associated changes in the gut microbial environment, opening new horizons for the development of non-invasive strategies for CRC screening [45,46].

Although several studies have investigated microbiota components associated with sporadic CRC [47], little is known about the role of the gut microbiota in hereditary CRC conditions.

With this review, we are going to provide insights into the latest data available about the potential of the human gut microbiota as a CRC diagnostic tool, with a particular emphasis on the few available studies that have linked hereditary CRC syndromes with the microbiota.

## 2. Hereditary and Sporadic Colorectal Cancer and Their Link to the Human Gut Microbiota

The Knudson’s classic two-hit model was hypothesized to explain CRC carcinogenesis and it seems to be in agreement with a possible role of microorganisms in oncogenic mutation insurgence [48,49]. In this model, the colorectal carcinogenesis begins with one mutation in each allele of the *APC* gene; subsequently, additional mutations in further genes could contribute to CRC development. Both hereditary and spontaneous CRC development present this pattern [49].

The gut microorganisms could produce virulence factors and secondary metabolites that could contribute to carcinogenesis provoking mechanisms such as DNA damage, DNA methylation, chromatin structure and non-coding RNA expression in colon epithelial cells [48].

In this context, researchers are looking for possible gut microorganisms able to interact with colon epithelial cells causing specific mutations that contribute to CRC development, taking advantage of modern molecular technologies (i.e., next-generation sequencing (NGS)).

One of the most studied pathogens involved in colorectal carcinogenesis is the *pks*+ *Escherichia coli* strain, which produces the colibactin toxin able to induce DNA double-strand breaks, chromosomal aberrations, and cell cycle arrest in cells in vitro [48,50,51]. Recently, Pleguezuelos-Manzano et al. demonstrated the correlation between *pks*^+^ *E. coli* strains producing colibactin and the occurrence of oncogenic mutations [51]. In vitro, colibactin can alkylate DNA on adenine residues and causes double-strand breaks. The authors treated human intestinal organoids by repeated luminal injections of genotoxic *pks*+ *E. coli* cultures for about five months. In these organoids they found a distinct mutational signature also detected in 5876 human cancer genomes from two independent cohorts, in particular with CRC, thus correlating the carcinogenic mutations to the presence of colibactin produced by *E. coli* [51].

Another pathogen extensively investigated for its role in CRC development is the enterotoxigenic *Bacteroides fragilis* strain (ETBF). ETBF produces a zinc-dependent metalloprotease toxin (BFT) that targets epithelial tight junctions through binding to an unidentified colon epithelial cell receptor via γ-secretase-dependent signal transduction [52,53]. In a murine model, Chung et al. demonstrated that BFT initiates and/or promotes CRC by triggering a pro-carcinogenic multi-step inflammatory cascade requiring IL-17R, NF-κB, and Stat3 signaling in colonic epithelial cells [54].

Deja et al. studied the colonic mucosa of FAP patients and identified bacterial biofilms composed predominantly of both *pks*^+^ *E. coli* and enterotoxigenic *B. fragilis*. By infecting mice with both strains, they detected increased interleukin-17 in the colon and DNA damage in colonic epithelium with early neoplasia of the colon [55].

In some cases, microbial virulence factors can induce chronic inflammation of host tissue, stimulating cellular proliferation contributing to CRC development.

For instance, a high level of *Fusobacterium nucleatum* was found in human colorectal adenomas and carcinomas [56]. *F. nucleatum* produces the adhesion virulence factor FadA that could help the pathogen to enter human epithelial cells, stimulate oncogenic gene expression and promote growth of CRC cells. Furthermore, *F. nucleatum* synthesizes Fap2, an autotransporter protein, which is able to potentiate the CRC progress by inhibiting immune cell activity [56,57].

It was demonstrated that *F. nucleatum* promotes tumorigenicity in an APC^Min/+^ mouse model, carrying *APC* mutations with a consequent predisposition to multiple intestinal neoplasia [58,59]. It was shown that, in mouse models, *F. nucleatum* regulates miR21 expression through the TLR4/MYD88/NFκB pathway, and it correlates with high expression of miR21 and poor clinical outcomes in human CRC tissues [58]. The pathogenic role of *F. nucleatum* in CRC patients was also investigated [60,61], suggesting that *F. nucleatum*, such as *pks*^+^ *E. coli* and enterotoxigenic *B. fragilis* may be targeted for CRC prevention and treatment [62].

Not only bacterial virulence factors, but also specific microbial metabolites, such as butyrate, were shown to severely affect CRC development [63,64,65,66], with the metabolome being a dominant factor responsible for variations in the gut microbiota communities in CRC [67]. These shifts in the CRC tumor microenvironment facilitate the assembly of a microbial community that is specific for CRC late-stage tumor, supporting a “driver–passenger” conceptual model, where CRC driver bacteria possess specific oncogenic properties that may drive tumorigenesis, while CRC passenger bacteria respond to changes in the tumor environment and are thus enriched in CRC tumor tissue [68,69]. It has been recently demonstrated that the catabolism of microbial metabolites like butyrate can induce the production of reactive oxygen species, the accumulation of the 8-oxo-7,8-dihydro-2′-deoxyguanosine lesions and double-strand DNA breaks in MMR-deficient cells [63,70]. This will lead to further accumulation of mutations that will eventually drive neoplasia. LS patients, harboring mutations in MMR genes, represent approximately 5% of all CRC cases [71], and an additional 15% of sporadic CRC patients carry mutations or have epigenetically silenced MMR genes, thus highlighting the risk associated to DNA damage induced by pathogens and gut-associated microorganisms [63]. A separate study showed that, in a genetic model of CRC (i.e., genetically sensitized *MSH2* and *MLH1* -deficient mice), the gut microbiota stimulated polyp formation by providing butyrate that induced hyper-proliferation and transformation of colon epithelial cells, suggesting a role of butyrate-producing gut microbes in the etiology of CRC [72]. In this mouse model, there was no evidence that the microbiota produced genotoxic agents [72].

*Faecalibacterium*, *Roseburia*, *Butyrivibrio*, and commensal species of Clostridia are all butyrate-producing bacteria [73,74]. Interestingly, *F. nucleatum*, which was previously indicated as one of the pathogens involved in CRC development, is a butyrate producer associated with CRC that releases ammonia as a by-product of its butyrate pathways [74]. It has been shown that higher concentrations of ammonia in the gut could increase CRC risk and possibly promote the presence of pathogenic bacteria like *Escherichia* and *Enterococcus* in the gut [75,76,77]. Carbohydrates have been implicated in the etiology of MMR-deficient CRC, and butyrate represents the principal carbohydrate-derived metabolite [72]. It has been demonstrated that the ablation of specific members of the gut microbiota, using different antibiotic treatment regimens, lead to a 75% reduction in polyp numbers in MMR-deficient mice, and butyrate was the only metabolite that was significantly diminished [72]. The monitoring of treatment effects on the gut microbiota showed that all of the treatments lead to a reduction of Firmicutes, the major butyrate-producing bacteria [78].

It has been hypothesized that metabolic alterations in a CRC environment can confer specific growth advantage in the CRC gut-associated microbiota [79]. This evidence supports the so-called driver–passenger conceptual model, where CRC driver bacteria possess specific oncogenic properties that may drive tumorigenesis, while CRC passenger bacteria respond to changes in the tumor environment and are thus enriched in CRC tumor tissue [68]. For example, *F. nucleatum* has been shown to specifically benefit from CRC metabolites (e.g., alanyl-histidine, pantothenic acid, meso-2-6-diaminoheptanedioate, nicotinic acid) [68,79]. However, because *F. nucleatum* is also hypothesized to drive tumorigenesis via its FadA virulence factor, it might be regarded as driving passenger bacterium.

Overall, these last findings underline the fundamental role of some bacterial pathogens to induce colorectal carcinogenesis and stimulate the study of other possible gut-associated bacteria able to contribute to CRC development.

## 3. Microbial Interaction with Intestinal Immune Responses

Host genetic susceptibility and environmental factors play an important role in the aberrant interactions between the microbiota and the host’s immune system, thus negatively impacting cancer immune surveillance [80]. For example, dysbiosis and immune dysfunctions may allow increased bacterial translocation due to altered barrier function, adenoma invasion by microbial products and the induction of cytokines, such as IL-17 and IL-23, maintaining an inflammatory environment within the CRC tissue [81,82,83]. Inflammation might in turn promote tumorigenesis by altering gut microbial communities and promoting the growth of genotoxic bacteria [84].

While several studies have investigated the human immune responses in CRC [85,86,87,88,89,90,91,92], what we know about the interplay between immune responses and gut microbiota in CRC is still very limited [92].

In sporadic CRC, correlations have been made between gut microorganisms, intestinal permeability and inflammation [93,94,95,96].

Tetz and collaborators studied the effects of exposure to a bacteriophage cocktail on intestinal permeability and gut microorganism’s abundance using a rat model [97]. They found higher levels of plasma lipopolysaccharide (LPS), related to impaired gut permeability, and increased serum concentrations of tumor necrosis factor-alpha (TNF-α), interleukin (IL)-1β, and IL-6. The bacterial genera *Blautia*, *Catenibacterium*, *Lactobacillus*, and *Faecalibacterium* were reduced, while an increase in *Butyrivibrio*, *Oscillospira* and *Ruminococcus* was detected [97].

More recently, a study showed that gut microbial imbalances and “leaky gut” conditions in CRC patients lead to a dysregulated inflammatory biomarker profile, with circulating levels of LPS being significantly elevated in CRC patients [93]. Furthermore, the association between LPS levels, inflammation, and hematological dysfunction suggested that chronic inflammation and an activated coagulation system could promote the tumorigenesis process [93]. It is thus possible to speculate that blood-based inflammatory markers could be used in combination with CRC screening for CRC diagnosis.

An increase in intestinal permeability and the inflammatory response driven by IL-6 was also observed during microbial formation of biofilms in the early stage of CRC development [96]. It is noteworthy that, in FAP patients, examination of biofilms attached to the colonic mucosa showed a predominance of *E. coli* and *B. fragilis*, possibly increasing CRC risk in patients with hereditary CRC conditions [55]. In sporadic CRC, it was shown that the *B. fragilis* ETBF strain produces the toxin fragylisin, a metalloproteinase increased in patients with advanced CRC that is responsible for increasing mucosal permeability, enhancing IL-17 cytokine secretion and STAT3 activation [52].

Other studies linked the gut microbiota to a modulated gut inflammation and carcinogenesis. For example, Yu et al. have recently demonstrated in a murine model that gut dysbiosis can lead to increased susceptibility to inflammation associated with CRC process via the induction of pro-inflammatory CD8^+^ IFNγ^+^ T cells, which can lead to increased T cell depletion within the CRC microenvironment [94].

In hereditary CRC conditions, very few data are available. It has been shown that pre-malignant lesions of LS patients display immune system activation well before cancer development [98]. In these patients the immune activation precedes the development of mutations and they are characterized by higher expression of specific markers for immune activation, such as CD4^+^ T cells, pro-inflammatory molecules, and checkpoint proteins such as programmed cell death 1 ligand (1PD-L1) and lymphocyte activation gene 3 [98]. This immune profile was independent of mutational rate, neoantigen formation, and MMR status [98].

Because of their role in pathogenicity of gastrointestinal diseases [99], CD4^+^ T cells have been described as a valuable therapeutic target [100]. Gut commensal bacteria can modulate the production of pro- and anti-inflammatory CD4^+^ T cells, such as T helper 17 (Th17) and colon-resident regulatory T cells [101]. It can thus be speculated that the early immune activation observed in LS patients might be triggered by patient’s gut microbiota composition, influenced by environmental factors such as diet and lifestyle. Because ETBF was shown to be necessary for synergistic tumor induction under co-colonization conditions with *pks*^+^ *E. coli* in azoxymethane–treated interleukin-10 (IL-10)–deficient mice [55], the role of IL-17 was tested in a co-colonized azoxymethane mouse model [55]. It was demonstrated that *pks*^+^ *E. coli* and ETBF accelerated colon tumorigenesis and reduced survival. This was associated with an IL17-mediated increase in colon inflammation that was necessary, but not sufficient, for tumorigenesis, as robust IL-17 induction by ETBF mono-colonization only induced scanter colon tumorigenesis [55].

Interestingly, it has been shown that introduction of altered Shaedler Flora species in mice induces Treg cells in a strain-dependent manner [102], thus suggesting that microbiota effects on T cell differentiation are dependent on the host’s genetic background [100].

The gut microbiota plays a major role in the modulation of intestinal and extra-intestinal immune diseases [100,103,104,105]. As intestinal immune homeostasis is dependent on a balanced microbiota composition [106], it appears clear that intestinal dysbiosis might be responsible for promoting immune imbalances and inflammation [100]. It is noteworthy that there are no data regarding the microbiota composition of CRC sporadic or hereditary patients and its impact on the human mucosal immune environment. Additional work to correlate the human microbiota and the associated mucosal immune responses in carcinogenesis is urgently needed.

## 4. Microbial Biomarkers in Sporadic and Hereditary CRC

Specific bacteria were found to be enriched in CRC patients and their potential as gut microbiota biomarkers in CRC screening has been described [107]. Enriched bacterial genera and species in sporadic CRC patients have been extensively reviewed [47]. In this case, the gut microbiota is characterized by a lower abundance of potentially protective bacteria (for example, *Bifidobacterium*), and an increased abundance of pro-carcinogenic microorganisms (such as *Bacteroides*, *Escherichia*, *Fusobacterium*, *Porphyromonas*, etc.) [38,51,52,108]. These pro-carcinogenic bacteria showed increased abundance in fecal and mucosal samples from sporadic CRC patients and have been extensively characterized (Table 2).

Taking advantage of it, these microorganisms could be used as CRC biomarkers. For these reasons, their study as well as the set-up of methods used for their quantification and their detection are mandatory for medicine progress.

**Table 2:** Microbial signatures in sporadic CRC.

Pro-Carcinogenic Bacteria	Sample Specimen	Molecular Method	CRC Stage	References
Proteobacteria and Firmicutes	mucosa	16S rRNA sequencing	adenoma-adjacent tissue	[109]
*Atopobium parvulum, Actinomyces odontolyticus*	stools	metagenomic studies	low-risk adenomas, high-risk adenoma	[108]
*Erysipelotrichaceae*	stools	16S rRNA sequencing	high risk adenoma	[110]
*Bacteroides dorei, Bacteroides massiliensis*	stools	metagenome-wide association study	advanced adenoma	[111]
*Fusobacterium nucleatum, Solobacterium moorei*	stools	metagenomic studies	high-risk adenoma, CRC stages	[108]
*Fusobacterium, Bacteroides fragilis, Gemella, Peptostreptococcus, Parvimonas, Granulicatella.*	mucosa	16S rRNA sequencing	carcinoma	[109]
*F. nucleatum, Peptostreptococcus stomatis, Parvimonas micra, S.moorei.*	stools	metagenomic studies	carcinoma	[46]
*B. massiliensis, Bacteroides ovatus, Bacteroides vulgatus, Escherichia coli*	stools	metagenome-wide association study	carcinoma	[111]
*Porphyromonas assaccharolytica, P. stomatis, P. micra, F. nucleatum*	stools	16S rRNA sequencing	carcinoma	[112]
*Sutterella, Escherichia/Shigella*	stools	16S rRNA sequencing	carcinoma	[110]
*Coprococcus, Prevotella, Fusobacterium, Parvimonas, Peptostreptococcus*	mucosa	16S rRNA sequencing	carcinoma, adenomas/polyps	[113]
*Alistipes, Akkermansia, Halomonas, Shewanella*	stools	16S rRNA sequencing	rectal and distal cancers	[113]
*Faecalibacterium, Blautia, Clostridium*	stools	16S rRNA sequencing	proximal cancer	[113]
*P. micra, Parvimonas spp., F. nucleatum, Gemella morbillorum, P. stomatis, Porphyromonas, Porphyromonas uenonis, Porphyromonas somerae, Porphyromonas asaccharolytica, Prevotella intermedia, Prevotella nigrescens, Clostridium symbiosum, Clostridium bolteae/clostridioforme, Dialister, S. moorei, Hungatella hathewayi, Ruminococcus torques, Anaerococcus obesiensis/vaginalis, Anaerotruncus, Subdoligranulum.*	stools	shotgun metagenomic studies	carcinoma	[114]
*pks^+^ E. coli, fadA^+^ F. nucleatum*	stools	shotgun metagenomic studies	carcinoma	[114,115]
*Erysipelotrichaceae*	stools	16S rRNA sequencing	hyperplastic polyps (serrated pathway)	[110]
*Streptococcus bovis/gallolyticus*	-	-	colorectal tumor tissues	[116]
*Streptococcus* sp. strain VT 162	saliva	WGS	-	[117]
**Bacteria with Protective Action**				
*Lachnospiraceae incertae sedis, Coprococcus*	stools	16S rRNA sequencing	-	[113]
Lactic acid-producing commensals (*Bifidobacterium animalis, Streptococcus mutans, Streptococcus thermophilus*)	stools	metagenome-wide association study	-	[111]
*Lachnospiraceae*	stools	16S rRNA sequencing		[110]

### 4.1. Focus on Hereditary CRC

Little data are available regarding the role of the gut microbiota in hereditary CRC syndromes. This is concerning as patients affected by genetic conditions, such as LS, are at a much greater risk of cancer than the general population. One of the few studies available in literature about the characterization of the gut microbiota in relation to LS patients reported enrichment of *B. fragilis* and *Parabacteroides distasonis* species, and Pseudomonadaceae family, in LS patients [118]. Those specific enrichments were significant when LS patients were compared to non-mutation carriers, while differences between LS patients and MMR mutation carriers without CRC were not significant [118]. The lack of information regarding the methodologies applied in the study makes interpreting these results difficult and really does not add much weight to the argument of whether the microbiota is important in the development of cancer in LS patients. Moreover, causality was not established, as it is not clear whether the enrichment detected was causative of LS condition or just a consequence of the genetic mutation at the core of LS (Table 3).

In a recent study, the colonic biopsy and fecal microbiotas of patients with LS and their relationship with the development of colonic pre-neoplastic lesions was investigated [119]. Microbiota associated with patients’ adenomas were assessed at baseline and after 1 to 2 years of follow-up, finding that commensal bacteria with pathobiont-like behavior, broadly influencing whole-gut ecology and metabolism, were the ones associated with adenomas and/or disease progression. However, the authors detected some abundant species (prevalence >90%) in stool samples of LS patients, such as *Eubacterium rectale*, *Bacteroides uniformis*, and *Faecalibacterium prausnitzii* [119].

Previous findings confirmed these results. In particular, *F. prausnitzii* and *B. uniformis* were found to be over-represented in a cohort of LS patients, together with *P. distasonis*, *Ruminococcus bromii*, *Bacteroides plebeius and B. fragilis* [118,120].

These results support a model of CRC development where environmental factors such as diet, drive individuals’ microbiota components that can modulate patients’ early risk to cancer. Accordingly, to the driver–passenger theory, these microbiota components can be defined as bacterial drivers, while more specifically casual microorganisms, usually detected in later-stage tumors, represent passenger bacteria [121].

Clinical factors that are specific to LS patients, such as medications used, surgery type and gene mutations, are significantly associated with individual taxonomic members of the mucosal and/or stool communities [119] (Table 3).

Yan et al. reported individual microbes associated with Lynch-relevant clinical factors, with members of the Clostridiaceae family being significantly depleted in the stool microbiota of individuals with adenomas, while Lachnospiraceae and Ruminococcaceae significantly enriched [119]. Biopsy samples were enriched in *Methanobrevibacter*, while *Desulfovibrio* was enriched in both stool and biopsy samples [119]. Among the LS related clinical factors the authors investigated, the potential effects of specific mismatch repair genes (*MLH1, MSH2, MSH6, PMS2*) on the microbiota was described. *MLH1* and *MSH2* mutations carriers were characterized by a decrease in Clostridiales and an increase in *Blautia* and *Oscillospira* (only in *MLH1* mutation carriers) [119].

Common limitations of these studies include small cohorts in each LS-associated genotype, which impairs the ability to characterize associations between specific driver mutations and microbiota composition. Most importantly, microbiota associations have been investigated in either very-early-stage adenoma or after patients’ surgery, and no data are available for late-stage CRC in LS patients [119,120].

Ferrarese et al. (2020) analyzed fecal and oral samples from LS patients after about 9.2 years of their previous cancer surgery. They found an increase of Bacteroidetes and Proteobacteria and a decrease of Firmicutes at the phylum level and of Ruminococcaceae at the family level in LS fecal samples. As the authors suggest, their results are similar to those observed in sporadic CRC; however, a larger cohort of patients needs to confirm this [122].

**Table 3:** Overview of human studies investigating the role of microbiota in the context of hereditary CRC syndromes.

Biological Sample—Time Point(s) for Collection	Controls	Mutation (n)	Location	Analysis	Main Findings	References
LS patients mucosal and feces microbiome samples—at colonoscopy prior to colorectal adenoma development and follow-up surveillance colonoscopy (1 to 2 years) (n = 100)	-	*MLH1* (30), *MSH2* (32), *MSH6* (23), *PMS2* (13), Unknown (a diagnosis of LS) (2)	Clinical and Translational Epidemiology Unit, Massachusetts General Hospital and Harvard Medical School, Boston and Department of Medicine, Memorial Sloan Kettering Cancer Center, NY, USA	Metagenomics, Metatranscriptomics and 16S rRNA sequencing	*Eubacterium rectale*, *B. uniformis* and *F. prausnitzii* were the most abundant in fecal samples; *Bacteroides, Faecalibacterium*, and *Ruminococcus* were the most abundant across mucosal biopsies	[46]
Feces microbiota samples of LS female patients who had developed either gynecological cancer only or colonic lesions, with/without additional tumors—2 years after cancer surgery (n = 10)	Healthy females, without family history of cancer (n = 8)	*MSH6* (7), *MLH1* (1), *MSH2* (1), *PMS2* (1)	Varese Hospital (Ospedale di Circolo di Varese-ASST dei Sette Laghi), Italy	16S rRNA sequencing	*F. prausnitzii, P. distasonis, R. bromii, B. plebeius, B. fragilis*, and *B. uniformis* were over-represented in LS patients	[47]
Feces microbiota samples of members from 14 LS families, including patients with CRC, MMR mutation carriers without CRC, and non-mutation carriers (tot n = 73)	-	-	-	16S rRNA sequencing	*B. fragilis, P. distasonis* and Pseudomonadaceae were associated with LS CRC.	[45]
Feces (17) and salivary (37) microbiota samples from LS patients after about 9.2 years of cancer surgery	Subjects without a history of CRC, inflammatory bowel diseases, metabolic syndrome, with PREMM5 < 2.5%	17 LS patients; 2 (*MLH1*), 14 (*MSH2*) 1 (*MSH6*). 37 LS patients: 9 (*MLH1*), 23 (*MSH2*) 2 (*MSH6*), 3 (*PMS2*) MLH1.	IRCCS Ospedale San Raffaele Scientific Institute, 20132 Milan, Italy	16S rRNA sequencing	An increase of Bacteroidetes and Proteobacteria and a decrease of Firmicutes at the phylum level and of Ruminococcaceae at the family level.	[122]
Polyps and paired normal tissues were collected from FAP patients undergoing colon surgery (n = 25)	Subjects without a history of CRC, inflammatory bowel disease, or antibiotic usage within three months (n = 23)	*APC* (3), *MYH* (1), Unknown (a diagnosis of LS) (21)	Johns Hopkins Hospital, Baltimore, MD, USA	FISH, Microculture	Biofilms comprised primarily of *E. coli* and *B. fragilis*	[59]
Biopsy and stool samples from LS patients with early adenomas (n = 100)	-	*MLH1* (30), *MSH2* (32), *MSH6* (23), *PMS2* (13), Unknown (a diagnosis of Lynch syndrome) (2)	Center for Cancer Risk Assessment and Division of Gastroenterology, Massachusetts General Hospital (MGH)/Clinical Genetics and/or Gastroenterology Services, Memorial Sloan Kettering Cancer Center (MSKCC)	Metagenomics, Metatranscriptomics and 16S rRNA sequencing	Decrease in order Clostridiales (*MLH1* and *MLH2* mutation carriers), increase in *Blautia* and *Oscillospira* (*MLH1* mutation carriers). *Clostridium* enriched in subjects with subtotal colectomy, *Prevotella, Lachnospira, Ruminococcus* and *Megasphaera* enriched in subjects with left colectomy. *Fusobacterium* and *Ruminococcus* enriched in right-sided colectomies. *Subdoligranulum* genus, *Barnesiella intestinihominis*, and *Alistipes shahii* species reduced in left colectomies.	[119]

In patients with sporadic CRC, a pro-oncogenic state is correlated with the formation of biofilms in the normal mucus layer [123,124]. Biofilm formation triggers mucosal inflammation response by breaching into the colonic mucus layer, thus affecting bacterial–epithelial cell contact [123,125]. The potential role of biofilm formation in patients with FAP has been investigated by Dejea et al., where the mucosa of FAP patients at clinically indicated colectomy was examined [55]. In this study, patchy biofilms were found to be invading the mucus layer, with *E. coli* and *B. fragilis* being the main bacteria scattered along the entire length of the colon [55]. These results were confirmed in a mouse model of FAP driven by APC mutations, suggesting that APC mutations may promote mucosal bacterial adherence [55].

Strong experimental evidence supports the carcinogenic potential of molecular subtypes of *pks*^+^ *E. coli* and *B. fragilis* in CRC [52,89,123] and reinforces what was subsequently found in the mucosal tissue of FAP patients, where approximately 60% to 68% of mucosal tissue cultures were populated by *pks*^+^ *E. coli* or ETBF, compared with 30% and 22% of healthy controls, respectively. Moreover, both BFT and colibactin producing *E. coli* were present in the mucus layer of FAP biofilms in direct contact with the FAP epithelium [55]. In FAP mouse models, co-colonization with *pks*^+^ *E. coli* and ETBF enhanced DNA damage in the colonic epithelium and it was associated with an IL17-mediated increase in colon inflammation, which contributed to an accelerated colon tumorigenesis [55].

These findings suggest that tumorigenic bacteria may promote early tumorigenesis in FAP patients and may thus represent good biomarkers in hereditary CRC.

Due to the difficulty in studying hereditary CRC syndromes, a clinical trial is in progress entitled “Metagenomic Evaluation of the Gut Microbiome in Patients with Lynch Syndrome and Other Hereditary Colonic Polyposis Syndromes (NCT02371135)” [126,127]. In this study, the role of gut microbiota on CRC development in 225 individuals with LS or other polyposis syndromes is under evaluation. Researchers are collecting stool specimens and/or colon tissue samples from people with LS and other hereditary colonic polyposis syndromes. Information about diet and lifestyle of participants is being collected. The samples collected will be used to study the role of gut bacteria in CRC risk, in addition to the hereditary risk and potential dietary impact [126,127].

It is noteworthy that late CRC diagnosis is associated with a 5-year survival rate of <10% [128]. In this context, the gut microbiota can be regarded as a source of non-invasive diagnostic and prognostic marker that could help in the early detection of CRC, thus increasing the patients’ survival rate to >85% [129]. While some bacterial species that can accurately discriminate between sporadic CRC cases and controls have already been identified [31], there is still a big gap in the identification of potential bacterial biomarkers for the potential development of a non-invasive screening method for CRC patients affected by hereditary syndromes.

### 4.2. Fungal and Viral Contribution to CRC Development

The significant contribution of gut microorganisms in CRC pathogenesis is widely accepted by now. However, most studies have focused on the role of gut bacteria, underestimating the role of another class of commensals, namely the mycobiota, might also be involved in CRC development [130]. Two recent studies strongly support the role of mycobiota in colitis-associated CRC [131,132]. Although these two studies used the same gene-targeted mouse model, the Card9-deficient mice were susceptible to diverse fungal infections. The microbiota difference detected in their mouse models makes the effect of the mycobiota strongly dependent on mouse microbial composition [130]. This further sustains the important role of environmental factors, such as diet, on microbiota composition.

Previously, the gut mycobiota of mucosal samples of patients with adenomas at different stages of disease development was investigated [133]. It was shown that fungal diversity was lower on adenomas compared to other areas of the colon, with two pathogenic fungi, *Candida* and *Phoma*, being over-represented in all of the biopsy samples. Moreover, two other pathogenic fungi, *Fusarium* and *Trichoderma*, were enriched in advanced-adenomas samples, compared to non-advanced subjects and adjacent biopsy samples [133]. It is thus possible that these pathogenetic fungi are predominantly present in mucosa biopsy samples of adenoma patients at different stages of adenoma progression, therefore potentially representing diagnostic biomarkers.

A more recent study confirmed the presence of different mycobiome signatures in fecal samples of patients with early- and late-stage CRC [134]. In this study, the authors identified CRC-specific mycobiota components, such as *Rhodotorula* and *Malassezia* of the Basidiomycota phylum and *Acremonium* of the Ascomycota phylum, both reported as opportunistic pathogens [134,135,136]. Interestingly, the fungal species *Saccharomyces cerevisiae*, a major component of the human gut microbiota, was found to be depleted in CRC, suggesting a beneficial role of *S. cerevisiae* in the gut and a potential therapeutic approach for CRC prevention or treatment [134]. Of importance, this study showed that the bacteria-fungi correlation network is altered in CRC patients compared to healthy controls. For instance, classes of Ascomycota phylum correlated synergistically with the bacterial Proteobacteria phylum in control patients, while additional fungal classes of Basidiomycota and Mucoromycota phyla were observed to participate in new interkingdom interactions in CRC subjects [134]. Also, correlation networks within fungi became more positive in CRC, while those between fungi and bacteria were largely negative [134]. Future investigations characterizing the functional consequences of an altered mycobiome composition and the role of bacteria–fungi interactions in CRC will further demonstrate the potential of fungal biomarkers for CRC screening. To date, nothing is present about the study of the gut mycobiome in relation to hereditary CRC disorders.

The gut is also a consortium of prokaryotic- (bacteriophages) and eukaryotic viruses including both DNA and RNA viruses. Shkoporov et al. demonstrated the presence in the gut of a persisting fraction of the viral community, mainly consisting of virulent bacteriophages targeting the major taxonomic groups of gut bacteria [137]. These virulent phages can lyse and reduce the gut bacterial component; consequently, the opportunistic pathogens could take advantage by invading the tissue and contributing to the development of CRC [138].

Hannigan et al. showed that the gut virome was altered in CRC patients relative to those with healthy people. The authors speculated that the phages could alter and lyse bacterial microbiota composition giving the opportunity to opportunistic bacteria (such as *F. nucleatum*) to colonize the gut epithelium driving CRC progression. In this way, these bacteria could induce the transformation of the epithelial cells, starting an inflammatory immune response [139].

## 5. The Human Gut Microbiota as CRC Predisposing Factor: Therapeutic Potential

During the last decade, understanding of the molecular mechanism of host genetics role in CRC has remarkably progressed, and variation in the gut microbiota communities has been linked to host genetic variation in CRC [140]. Nevertheless, many questions about the missing link of microbiota dysbiosis and genetic polymorphism in CRC hereditary conditions remain unanswered.

Does dysbiosis drive CRC by inducing DNA damage and does it induce tumorigenesis for those who are the carriers of DNA germline variants? In this context, the homeostasis on the gut microbiota is of great importance.

The consumption of microorganisms that are considered to be “healthy” for human gut (i.e., probiotics) has had some success in managing gastrointestinal diseases, such as irritable bowel syndrome [141]. Recently, the potential therapeutic role of microbiota organisms in sporadic CRC prevention and treatment has been extensively revised [142].

The promotion of gut microbial homeostasis through the use of probiotics (i.e., live microorganisms that when administered in adequate amounts confer a health benefit on the host [143]) may help in restoring a healthy gut microbiota, thus in turn promoting successful CRC therapy through improved sensitivity to chemo drugs or immune checkpoint inhibitors (ICIs) [144].

For instance, gut microbial dysbiosis, induced by antibiotic treatments, significantly reduced the anti-tumor efficacy of 5- Fluorouracil treatment in a CRC mouse model [145]. However, probiotics supplementation with *Lactobacillus* and *Bifidobacterium*, the most common species found in the human gut, did not significantly increase 5- Fluorouracil activity [145]. Of interest, *Lactobacillus* daily oral administration resulted in reduced frequency of severe 5- Fluorouracil based chemotherapy related diarrhea in CRC patients [146].

In animal models of CRC the results are often inconsistent, reflecting the way in which gut dysbiosis is induced, probiotic supplements are used and probiotics are administered. Given the unclear rationale to translate animal studies to humans, some clinical trials are in progress regarding the use of probiotic strains to improve anticancer therapy. A clinical trial (NCT03782428) was performed to determine the effect of probiotic consumption containing six viable bacteria belonging to *Lactobacillus* and *Bifidobacterium* species for six months in CRC patients. The use of probiotics resulted in a decline in pro-inflammatory cytokines [127,147].

Otherwise, the number of clinical trials investigating probiotics in CRC prevention is scarce, the results are inconsistent and conclusions regarding probiotic efficacy difficult to draw [148].

For instance, *Lactobacillus* and *Bifidobacterium* have shown positive outcomes when administered to patients with sporadic CRC (revised in [149]). Furthermore, it was recently shown that *Clostridium butyricum*, which is a butyrate-producing bacterium, can inhibit CRC development by modulating Wnt signaling both in a murine model and in tumor colonic cells [150].

However, other probiotic species have shown no positive effects in either sporadic or hereditary CRC patients [151,152].

Studies investigating the best combination of different probiotics strains to be used for preventing CRC or reducing the CRC treatments side effects are still required, and the tailoring of a probiotic strategy to high-risk hereditary CRC syndrome carriers is highly warranted.

The contribution of gut microbiota to CRC immunotherapy seems to give more promising results [142,153,154,155]. ICIs modulate innate immunity and activate anti-tumor immune response, and the tumor environment (e.g., the microbiota) critically conditions therapy efficacy [142].

Recently, it has been demonstrated that the gut microbial metabolite inosine is produced by a commensal *Bifidobacterium* species and significantly enhances efficacy of anti-cytotoxic T-lymphocyte-associated antigen 4 therapy in CRC mouse models, by boosting conventional dendritic cells -dependent TH1 cell response [156]. As these ICIs-promoting bacteria are also found in humans, this finding has a high potential for translation into clinical practice [157].

A growing amount of evidence reports that checkpoint proteins, such as PD-1 and its ligands (PD-L1/PD-L2), promote tumor growth and metastasis and increase the function of immunosuppressive regulatory T cells [158,159]. Given the higher expression of checkpoint proteins such as 1PD-L1 in LS patients [98], it can be speculated that LS patients are the ones who would benefit the most from exploiting the application of the gut microbiota as a way to enhance ICIs efficacy.

ICIs are approved for use in mismatch repair–deficient cancers of any histology [160]. Inactivation of the DNA MMR pathway is followed by somatic mutations driving tumorigenesis in both sporadic and hereditary MSI CRC [161]. Notably, about 1300 somatic mutations are acquired in MSI CRC derived from LS patients, whereas an average of only 190 mutations are acquired in tumors with microsatellite stability [162]. Thus, patients with high MSI tumors represent a subgroup more likely to benefit from ICIs [161], and specific commensal gut bacteria have been implicated in the efficacy of ICIs [163]. Indeed, the commensal gut genus *Bifidobacterium* was associated with a clinical benefit for PD-L1 checkpoint blockade, and the use of antibiotics was shown to induce dysbiosis of gut microbiota with subsequent unresponsiveness to immunotherapy [164,165]. A recent study identified a consortium of 11 bacteria that act together to induce IFNγ+ CD8 T cells, enhanced resistance against pathogenic infections and improved the therapeutic efficacy of ICIs in mice [166]. Although these studies were not conducted in CRC models, they highlight the potential of microorganisms present in the gut microbiota as “immunotherapy probiotics”, thus helping patients in their response to immunotherapies.

The use of antibiotic treatment to fight gut dysbiosis and consequently to inhibit a possible CRC progression has been argued because it could also compromise immunotherapy efficacy or induce carcinogenesis by causing further microbial shifts.

Zackular et al. demonstrated that the treatment of an inflammation-based murine model of CRC with a cocktail of metronidazole, streptomycin, and vancomycin can affect the gut microbiota composition during the onset of inflammation and significantly decrease tumorigenesis process [167].

Not only can drug treatment contribute to induce gut dysbiosis, but in some cases it can kill gut pathobionts such as *B. fragilis* and other bacteria linked to inflammation and DNA methylation, suppressing tumor proliferation, invasion and growth [83]. Recently, metronidazole treatment of mice bearing a CRC xenograft reduced *F. nucleatum* cell load and cancer proliferation, suggesting the potential use of this antibiotic against *F. nucleatum*-associated CRC [168].

Antibiotic treatment is also considered a possible immunotherapeutic strategy, even if controversial data are available. The non-selective eradication of the commensal gut bacteria by antibiotics could abolish the antitumor immunity. Vétizou et al. demonstrated that an antibiotic cocktail, or imipenem alone, eradicated the immune response and restored tumor progression in CRC mouse models [154]. In agreement with this achievement, it has been recently reported that concomitant use of antibiotics and immunotherapy is associated with a high risk of disease progression in lung cancer patients [169].

Because of these controversial findings, the potential use of antibiotics in the prevention and treatment of CRC is still under investigation.

Recently, phage therapy has been considered as an alternative antimicrobial approach that has the advantage of being highly specific against a particular microbial pathogen. Dong and collaborators showed that a M13 phage, presenting silver nanoparticles on its surface capsid protein (M13@Ag) and binding to *F. nucleatum*, was able to selectively increase the host’s anticancer immune response both in vitro and in vivo [170]. Interestingly, M13@Ag combined with immune checkpoint inhibitors (α-PD1) or chemotherapeutics (FOLFIRI) prolonged mouse survival in a CRC murine model [170]. This approach is very promising because it does not induce drug resistance and it is selective against a specific microbial species.

The transfer of fecal microbiota from healthy individuals to patients is already successfully used against colitis caused by *Clostridium difficile* infection [171]. The major benefits of this procedure include modulation of immunotherapy efficacy and restoration of gut microbial eubiosis.

Recently, the effect of fecal microbiota transfer on FOLFOX chemotherapy regimen (5-fluorouracil, leucovorin, and oxaliplatin)-induced mucosal injury in BALB/c mice with adenocarcinoma was tested [172]. Fecal microbiota transfer reduced the severity of diarrhea and intestinal mucositis caused by chemotherapy treatment [172].

More studies are needed to assess the use of fecal microbiota transplantation to avoid side gut effects of chemotherapy treatments and to prevent and cure CRC.

## 6. Conclusions and New Perspectives

The gut microbiota is involved in many areas of human health and it is essential for life, providing nutrients and vitamins, protecting against pathogens and contributing to immune system development.

In these last years, great steps forward have been made regarding the study of gut microbiota and its contribution in CRC development, even if several questions are still unresolved. How could we understand the colon microbiota contribution to the pathogenesis of human sporadic and hereditary CRC? Does the total microbial community or the only presence of some bacteria influence colon carcinogenesis?

Currently, several studies associate human gut microbiota dysbiosis with CRC risk, but do not seem to reflect the complexity of this multifactorial disease.

Nowadays, there is still a gap in the knowledge of the full spectrum of microbial metabolites that are characteristic of the sporadic and hereditary CRC microenvironment, which is still unexplored.

The correlation present among human gut microbiota and a non-targeted, global metabolomics approach would possibly help the further development of CRC prognostic markers and therapeutic targets. By using the integrated –OMICS approach, researchers are trying to better understand this unknown field. In a recent study, Wang and collaborators found correlations between the abundance of some microbial taxa, butyrate-related gut metabolites, and DNA methylation. For instance, the increase of *Fusobacterium* abundance was correlated with a decrease in the level of 4-hydroxybutyric acid and expression of immune-related peptidase inhibitor 16 (PI16), Fc Receptor Like A and Lymphocyte Specific Protein 1. These findings increase our knowledge about the use potential of new metabolic gut biomarkers [173].

Similarly, Wilson and collaborators characterized the role of the carcinogenic colibactin, a metabolite produced by some *E. coli* strains, in CRC development [174]. They reported the introduction of a covalent DNA modification in human cell lines treated with colibactin-producing *E. coli*, showing that colibactin alkylates DNA [174]. This work could be useful for CRC diagnosis and demonstrates without doubt how a single gut microorganism may contribute to colorectal carcinogenesis [174].

It is noteworthy that some gut carcinogenic species can be involved in the development of both hereditary and sporadic CRC (e.g., *E. coli*, *B. fragilis*, and *F. nucleatum*). Further studies are ongoing to use this knowledge in clinical practice. If we are able to identify microbial biomarkers for CRC, we could then make an important impact by paving the way to new prevention strategies.

## Figures and Tables

**Table 1:** Leading risk factors of colorectal cancer.

Risk Factors		References
Hereditary factors	Hereditary colorectal cancer syndromes	[1], reviewed in [7,8]
	Positive family history	[1], reviewed in [7,8]
	**Contributing factors**	
Environmental factors	Pre-cancerous conditions	reviewed in [6]
	Elderly	[3], reviewed in [6]
	Male	[3], reviewed in [6]
	Smoking	[9]
	Large intake of red or processed meat	[10]
	Alcohol intake	[11]
	Microbial dysbiosis	reviewed in [12]
Other risk factors	Body fat and obesity	reviewed in [13]
	Type 2 diabetes	[14]
	Inflammatory bowel disease	reviewed in [15]
**Preventing Factors**		
	Physical activity	reviewed in [16]
	Whole grains	reviewed in [17]
	Dietary fiber	reviewed in [17]
	Fish intake	reviewed in [17]
	Vitamins (D, C, and others)	[18]
	Use of aspirin and other anti-inflammatory drugs	reviewed in [6]

## References

[1] International Agency for Research on Cancer, WHO (2018). Colorectal Cancer.

[2] Bray F., Ferlay J., Soerjomataram I., Siegel R.L., Torre L.A., Jemal A. (2018). Global cancer statistics 2018: GLOBOCAN estimates of incidence and mortality worldwide for 36 cancers in 185 countries. CA Cancer J. Clin..

[3] Arnold M., Sierra M.S., Laversanne M., Soerjomataram I., Jemal A., Bray F. (2017). Global patterns and trends in colorectal cancer incidence and mortality. Gut.

[4] Fitzmaurice C., Allen C., Barber R.M., Barregard L., Bhutta Z.A., Brenner H., Dicker D.J., Chimed-Orchir O., Dandona R., Dandona L. (2017). Global, Regional, and National Cancer Incidence, Mortality, Years of Life Lost, Years Lived with Disability, and Disability-Adjusted Life-years for 32 Cancer Groups, 1990 to 2015 A Systematic Analysis for the Global Burden of Disease Study. JAMA Oncol..

[5] Chan A.T., Giovannucci E.L. (2010). Primary Prevention of Colorectal Cancer. Gastroenterology.

[6] Dekker E., Tanis P.J., Vleugels J.L.A., Kasi P.M., Wallace M.B. (2019). Colorectal cancer. Lancet.

[7] Burt R.W., Disario J.A., Cannonalbright L. (1995). Genetics of Colon-Cancer—Impact of Inheritance on Colon-Cancer Risk. Annu. Rev. Med..

[8] Lynch H.T., Smyrk T.C., Watson P., Lanspa S.J., Lynch J.F., Lynch P.M., Cavalieri R.J., Boland C.R. (1993). Genetics, Natural-History, Tumor Spectrum, and Pathology of Hereditary Nonpolyposis Colorectal-Cancer—An Updated Review. Gastroenterology.

[9] Botteri E., Iodice S., Bagnardi V., Raimondi S., Lowenfels A.B., Maisonneuve P. (2008). Smoking and Colorectal Cancer a Meta-analysis. JAMA J. Am. Med. Assoc..

[10] Chan D.S.M., Lau R., Aune D., Vieira R., Greenwood D.C., Kampman E., Norat T. (2011). Red and Processed Meat and Colorectal Cancer Incidence: Meta-Analysis of Prospective Studies. PLoS ONE.

[11] Cai S.F., Li Y.J., Ding Y., Chen K., Jin M.J. (2014). Alcohol drinking and the risk of colorectal cancer death: A meta-analysis. Eur. J. Cancer Prev..

[12] Kyrgiou M., Kalliala I., Markozannes G., Gunter M.J., Paraskevaidis E., Gabra H., Martin-Hirsch P., Tsilidis K.K. (2017). Adiposity and cancer at major anatomical sites: Umbrella review of the literature. BMJ Brit. Med. J..

[13] Peeters P.J.H.L., Bazelier M.T., Leufkens H.G.M., de Vries F., De Bruin M.L. (2015). The Risk of Colorectal Cancer in Patients with Type 2 Diabetes: Associations with Treatment Stage and Obesity. Diabetes Care.

[14] Kim E.R., Chang D.K. (2014). Colorectal cancer in inflammatory bowel disease: The risk, pathogenesis, prevention and diagnosis. World J. Gastroenterol..

[15] Wong S.H., Yu J. (2019). Gut microbiota in colorectal cancer: Mechanisms of action and clinical applications. Nat. Rev. Gastroenterol. Hepatol..

[16] Shaw E., Farris M.S., Stone C.R., Derksen J.W.G., Johnson R., Hilsden R.J., Friedenreich C.M., Brenner D.R. (2018). Effects of physical activity on colorectal cancer risk among family history and body mass index subgroups: A systematic review and meta-analysis. BMC Cancer.

[17] Song M.Y., Chan A.T., Sun J. (2020). Influence of the Gut Microbiome, Diet, and Environment on Risk of Colorectal Cancer. Gastroenterology.

[18] Manson J.E., Cook N.R., Lee I.M., Christen W., Bassuk S.S., Mora S., Gibson H., Gordon D., Copeland T., D’Agostino D. (2019). Vitamin D Supplements and Prevention of Cancer and Cardiovascular Disease. N. Engl. J. Med..

[19] Syngal S., Brand R.E., Church J.M., Giardiello F.M., Hampel H.L., Burt R.W. (2015). ACG clinical guideline: Genetic testing and management of hereditary gastrointestinal cancer syndromes. Am. J. Gastroenterol..

[20] Yurgelun M.B., Kulke M.H., Fuchs C.S., Allen B.A., Uno H., Hornick J.L., Ukaegbu C.I., Brais L.K., McNamara P.G., Mayer R.J. (2017). Cancer Susceptibility Gene Mutations in Individuals With Colorectal Cancer. J. Clin. Oncol..

[21] Bonadona V., Bonaiti B., Olschwang S., Grandjouan S., Huiart L., Longy M., Guimbaud R., Buecher B., Bignon Y.J., Caron O. (2011). Cancer risks associated with germline mutations in MLH1, MSH2, and MSH6 genes in Lynch syndrome. JAMA.

[22] Dowty J.G., Win A.K., Buchanan D.D., Lindor N.M., Macrae F.A., Clendenning M., Antill Y.C., Thibodeau S.N., Casey G., Gallinger S. (2013). Cancer risks for MLH1 and MSH2 mutation carriers. Hum. Mutat..

[23] Broeke S.W.T., Brohet R.M., Tops C.M., Van Der Klift H.M., Velthuizen M.E., Bernstein I., Munar G.C., Lázaro C., Hoogerbrugge N., Letteboer T.G.W. (2015). Lynch syndrome caused by germline PMS2 mutations: Delineating the cancer risk. J. Clin. Oncol..

[24] Vasen H.F.A., Blanco I., Aktan-Collan K., Gopie J.P., Alonso A., Aretz S., Bernstein I., Bertario L., Burn J., Capella G. (2013). Revised guidelines for the clinical management of Lynch syndrome (HNPCC): Recommendations by a group of European experts. Gut.

[25] Kidambi T.D., Kohli D.R., Samadder N.J., Singh A. (2019). Hereditary Polyposis Syndromes. Curr. Treat. Options Gastroenterol..

[26] Leoz M.L., Carballal S., Moreira L., Ocana T., Balaguer F. (2015). The genetic basis of familial adenomatous polyposis and its implications for clinical practice and risk management. Appl. Clin. Genet..

[27] Sieber O.M., Lipton L., Crabtree M., Heinimann K., Fidalgo P., Phillips R.K.S., Bisgaard M., Orntoft T.F., Aaltonen L.A., Hodgson S.V. (2003). Multiple colorectal adenomas, classic adenomatous polyposis, and germ-line mutations in MYH. N. Engl. J. Med..

[28] Casper M., Spier I., Holz R., Aretz S., Lammert F. (2018). Phenotypic Variability of MUTYH-Associated Polyposis in Monozygotic Twins and Endoscopic Resection of A Giant Polyp in Pregnancy. Am. J. Gastroenterol..

[29] Lorca V., Rueda D., Martin-Morales L., Fernandez-Acenero M.J., Grolleman J., Poves C., Llovet P., Tapial S., Garcia-Barberan V., Sanz J. (2019). Contribution of New Adenomatous Polyposis Predisposition Genes in an Unexplained Attenuated Spanish Cohort by Multigene Panel Testing. Sci. Rep..

[30] Fearon E.R., Vogelstein B. (1990). A Genetic Model for Colorectal Tumorigenesis. Cell.

[31] Jones S., Chen W.D., Parmigiani G., Diehl F., Beerenwinkel N., Antal T., Traulsen A., Nowak M.A., Siegel C., Velculescu V.E. (2008). Comparative lesion sequencing provides insights into tumor evolution. Proc. Natl. Acad. Sci. USA.

[32] Sanchez J.A., Krumroy L., Plummer S., Aung P., Merkulova A., Skacel M., DeJulius K.L., Manilich E., Church J.M., Casey G. (2009). Genetic and epigenetic classifications define clinical phenotypes and determine patient outcomes in colorectal cancer. BJS.

[33] Pino M.S., Chung D.C. (2010). The Chromosomal Instability Pathway in Colon Cancer. Gastroenterology.

[34] Young J., Jenkins M., Parry S., Young B., Nancarrow D., English D., Giles G., Jass J. (2007). Serrated pathway colorectal cancer in the population: Genetic consideration. Gut.

[35] Bettington M.L., Chetty R. (2015). Traditional serrated adenoma: An update. Hum. Pathol..

[36] Ashktorab H., Kupfer S.S., Brim H., Carethers J.M. (2017). Racial Disparity in Gastrointestinal Cancer Risk. Gastroenterology.

[37] Shanahan F., Ghosh T.S., O’Toole P.W. (2020). The Healthy Microbiome (What Is the Definition of a Healthy Gut Microbiome?). Gastroenterology.

[38] Scott A.J., Alexander J.L., Merrifield C.A., Cunningham D., Jobin C., Brown R., Alverdy J., O’Keefe S.J., Gaskins H.R., Teare J. (2019). International Cancer Microbiome Consortium consensus statement on the role of the human microbiome in carcinogenesis. Gut.

[39] Brown J.M., Hazen S.L. (2018). Microbial modulation of cardiovascular disease. Nat. Rev. Microbiol..

[40] Maruvada P., Leone V., Kaplan L.M., Chang E.B. (2017). The Human Microbiome and Obesity: Moving beyond Associations. Cell Host Microbe.

[41] Sharon G., Sampson T.R., Geschwind D.H., Mazmanian S.K. (2016). The Central Nervous System and the Gut Microbiome. Cell.

[42] Kostic A.D., Xavier R.J., Gevers D. (2014). The Microbiome in Inflammatory Bowel Disease: Current Status and the Future Ahead. Gastroenterology.

[43] Buffie C.G., Bucci V., Stein R.R., McKenney P.T., Ling L.L., Gobourne A., No D., Liu H., Kinnebrew M., Viale A. (2015). Precision microbiome reconstitution restores bile acid mediated resistance to *Clostridium difficile*. Nature.

[44] Helmink B.A., Khan M.A.W., Hermann A., Gopalakrishnan V., Wargo J.A. (2019). The microbiome, cancer, and cancer therapy. Nat. Med..

[45] Zackular J.P., Rogers M.A.M., Ruffin M.T., Schloss P.D. (2014). The Human Gut Microbiome as a Screening Tool for Colorectal Cancer. Cancer Prev. Res..

[46] Yu J., Feng Q., Wong S.H., Zhang D., Liang Q.Y., Qin Y.W., Tang L.Q., Zhao H., Stenvang J., Li Y.L. (2017). Metagenomic analysis of faecal microbiome as a tool towards targeted non-invasive biomarkers for colorectal cancer. Gut.

[47] Ternes D., Karta J., Tsenkova M., Wilmes P., Haan S., Letellier E. (2020). Microbiome in Colorectal Cancer: How to Get from Meta-omics to Mechanism?. Trends Microbiol..

[48] Allen J., Sears C.L. (2019). Impact of the gut microbiome on the genome and epigenome of colon epithelial cells: Contributions to colorectal cancer development. Genome Med..

[49] Fearon E.R. (2011). Molecular Genetics of Colorectal Cancer. Annu. Rev. Pathol. Mech. Dis..

[50] Nougayrede J.P., Homburg S., Taieb F., Boury M., Brzuszkiewicz E., Gottschalk G., Buchrieser C., Hacker J., Dobrindt U., Oswald E. (2006). *Escherichia coli* induces DNA double-strand breaks in eukaryotic cells. Science.

[51] Pleguezuelos-Manzano C., Puschhof J., Huber A.R., van Hoeck A., Wood H.M., Nomburg J., Gurjao C., Manders F., Dalmasso G., Stege P.B. (2020). Mutational signature in colorectal cancer caused by genotoxic *pks*^(+)^
*E. coli*. Nature.

[52] Boleij A., Hechenbleikner E.M., Goodwin A.C., Badani R., Stein E.M., Lazarev M.G., Ellis B., Carroll K.C., Albesiano E., Wick E.C. (2015). The *Bacteroides fragilis* Toxin Gene Is Prevalent in the Colon Mucosa of Colorectal Cancer Patients. Clin. Infect. Dis..

[53] Sears C.L., Geis A.L., Housseau F. (2014). *Bacteroides fragilis* subverts mucosal biology: From symbiont to colon carcinogenesis. J. Clin. Investig..

[54] Chung L., Orberg E.T., Geis A.L., Chan J.L., Fu K., Shields C.E.D., Dejea C.M., Fathi P., Chen J., Finard B.B. (2018). *Bacteroides fragilis* Toxin Coordinates a Pro-carcinogenic Inflammatory Cascade via Targeting of Colonic Epithelial Cells. Cell Host Microbe.

[55] Dejea C.M., Fathi P., Craig J.M., Boleij A., Taddese R., Geis A.L., Wu X.Q., Shields C.E.D., Hechenbleikner E.M., Huso D.L. (2018). Patients with familial adenomatous polyposis harbor colonic biofilms containing tumorigenic bacteria. Science.

[56] Shang F.M., Liu H.L. (2018). *Fusobacterium nucleatum* and colorectal cancer: A review. World J. Gastrointest. Oncol..

[57] Lee S.A., Liu F., Riordan S.M., Lee C.S., Zhang L. (2019). Global Investigations of *Fusobacterium nucleatum* in Human Colorectal Cancer. Front. Oncol..

[58] Yang Y.Z., Weng W.H., Peng J.J., Hong L.M., Yang L., Toiyama Y., Gao R.Y., Liu M.F., Yin M.M., Pan C. (2017). *Fusobacterium nucleatum* Increases Proliferation of Colorectal Cancer Cells and Tumor Development in Mice by Activating Toll-Like Receptor 4 Signaling to Nuclear Factor-kappa B, and Up-regulating Expression of MicroRNA-21. Gastroenterology.

[59] Moser A.R., Pitot H.C., Dove W.F. (1990). A Dominant Mutation That Predisposes to Multiple Intestinal Neoplasia in the Mouse. Science.

[60] Castellarin M., Warren R.L., Freeman J.D., Dreolini L., Krzywinski M., Strauss J., Barnes R., Watson P., Allen-Vercoe E., Moore R.A. (2012). *Fusobacterium nucleatum* infection is prevalent in human colorectal carcinoma. Genome Res..

[61] Kostic A.D., Gevers D., Pedamallu C.S., Michaud M., Duke F., Earl A.M., Ojesina A.I., Jung J., Bass A.J., Tabernero J. (2012). Genomic analysis identifies association of *Fusobacterium* with colorectal carcinoma. Genome Res..

[62] Zeller G., Tap J., Voigt A.Y., Sunagawa S., Kultima J.R., Costea P.I., Amiot A., Boohm J., Brunetti F., Habermann N. (2014). Potential of fecal microbiota for early-stage detection of colorectal cancer. Mol. Syst. Biol..

[63] Irrazabal T., Thakur B.K., Kang M., Malaise Y., Streutker C., Wong E.O.Y., Copeland J., Gryfe R., Guttman D.S., Navarre W.W. (2020). Limiting oxidative DNA damage reduces microbe-induced colitis-associated colorectal cancer. Nat. Commun..

[64] Kim D.H., Jin Y.H. (2001). Intestinal bacterial beta-glucuronidase activity of patients with colon cancer. Arch. Pharm. Res..

[65] Saracut C., Molnar C., Russu C., Todoran N., Vlase L., Turdean S., Voidazan S., Copotoiu C. (2015). Secondary bile acids effects in colon pathology. Experimental mice study. Acta Cir. Bras..

[66] Russell W.R., Gratz S.W., Duncan S.H., Holtrop G., Ince J., Scobbie L., Duncan G., Johnstone A.M., Lobley G.E., Wallace R.J. (2011). High-protein, reduced-carbohydrate weight-loss diets promote metabolite profiles likely to be detrimental to colonic health. Am. J. Clin. Nutr..

[67] Louis P., Hold G.L., Flint H.J. (2014). The gut microbiota, bacterial metabolites and colorectal cancer. Nat. Rev. Microbiol..

[68] Tjalsma H., Boleij A., Marchesi J.R., Dutilh B.E. (2012). A bacterial driver-passenger model for colorectal cancer: Beyond the usual suspects. Nat. Rev. Microbiol..

[69] Geng J.W., Song Q.F., Tang X.D., Liang X., Fan H., Peng H.L., Guo Q., Zhang Z.G. (2014). Co-occurrence of driver and passenger bacteria in human colorectal cancer. Gut Pathog..

[70] Niles J.C., Wishnok J.S., Tannenbaum S.R. (2006). Peroxynitrite-induced oxidation and nitration products of guanine and 8-oxoguanine: Structures and mechanisms of product formation. Nitric Oxide.

[71] Lynch H.T., de la Chapelle A. (1999). Genetic susceptibility to non-polyposis colorectal cancer. J. Med. Genet..

[72] Belcheva A., Irrazabal T., Robertson S.J., Streutker C., Maughan H., Rubino S., Moriyama E.H., Copeland J.K., Surendra A., Kumar S. (2014). Gut microbial metabolism drives transformation of MSH2-deficient colon epithelial cells. Cell.

[73] Abbeele P.V.D., Belzer C., Goossens M., Kleerebezem M., De Vos W.M., Thas O., De Weirdt R., Kerckhof F.-M., Van De Wiele T. (2013). Butyrate-producing *Clostridium* cluster XIVa species specifically colonize mucins in an in vitro gut model. ISME J..

[74] Anand S., Kaur H., Mande S.S. (2016). Comparative In silico Analysis of Butyrate Production Pathways in Gut Commensals and Pathogens. Front. Microbiol..

[75] Richardson A.J., McKain N., Wallace R.J. (2013). Ammonia production by human faecal bacteria, and the enumeration, isolation and characterization of bacteria capable of growth on peptides and amino acids. BMC Microbiol..

[76] Corpet D.E., Yin Y., Zhang X.M., Remesy C., Stamp D., Medline A., Thompson L., Bruce W.R., Archer M.C. (1995). Colonic protein fermentation and promotion of colon carcinogenesis by thermolyzed casein. Nutr. Cancer.

[77] Xu J., Lian F., Zhao L., Zhao Y., Chen X., Zhang X., Guo Y., Zhang C., Zhou Q., Xue Z. (2015). Structural modulation of gut microbiota during alleviation of type 2 diabetes with a Chinese herbal formula. ISME J..

[78] Belcheva A., Martin A. (2015). Gut microbiota and colon cancer: The carbohydrate link. Mol. Cell. Oncol..

[79] Garza D.R., Taddese R., Wirbel J., Zeller G., Boleij A., Huynen M.A., Dutilh B.E. (2020). Metabolic models predict bacterial passengers in colorectal cancer. Cancer Metab..

[80] Zheng D.P., Liwinski T., Elinav E. (2020). Interaction between microbiota and immunity in health and disease. Cell Res..

[81] Irrazabal T., Belcheva A., Girardin S.E., Martin A., Philpott D.J. (2014). The Multifaceted Role of the Intestinal Microbiota in Colon Cancer. Mol. Cell.

[82] Jobin C. (2012). CRC—All about microbial products and barrier function?. Nat. Rev. Gastroenterol. Hepatol..

[83] Grivennikov S.I., Wang K.P., Mucida D., Stewart C.A., Schnabl B., Jauch D., Taniguchi K., Yu G.Y., Osterreicher C.H., Hung K.E. (2012). Adenoma-linked barrier defects and microbial products drive IL-23/IL-17-mediated tumour growth. Nature.

[84] Arthur J.C., Perez-Chanona E., Muhlbauer M., Tomkovich S., Uronis J.M., Fan T.J., Campbell B.J., Abujamel T., Dogan B., Rogers A.B. (2012). Intestinal inflammation targets cancer-inducing activity of the microbiota. Science.

[85] Pages F., Galon J., Fridman W.H. (2008). The essential role of the in situ immune reaction in human colorectal cancer. J. Leukoc. Biol..

[86] Pages F., Berger A., Camus M., Sanchez-Cabo F., Costes A., Molidor R., Mlecnik B., Kirilovsky A., Nilsson M., Damotte D. (2005). Effector memory T cells, early metastasis, and survival in colorectal cancer. N. Engl. J. Med..

[87] Galon J., Costes A., Sanchez-Cabo F., Kirilovsky A., Mlecnik B., Lagorce-Pages C., Tosolini M., Camus M., Berger A., Wind P. (2006). Type, density, and location of immune cells within human colorectal tumors predict clinical outcome. Science.

[88] Siddiqui S.A., Frigola X., Bonne-Annee S., Mercader M., Kuntz S.M., Krambeck A.E., Sengupta S., Dong H.D., Cheville J.C., Lohse C.M. (2007). Tumor-infiltrating Foxp3(-)CD4(+)CD25(+) T cells predict poor survival in renal cell carcinoma. Clin. Cancer Res..

[89] Wu S.G., Rhee K.J., Albesiano E., Rabizadeh S., Wu X.Q., Yen H.R., Huso D.L., Brancati F.L., Wick E., McAllister F. (2009). A human colonic commensal promotes colon tumorigenesis via activation of T helper type 17 T cell responses. Nat. Med..

[90] Qiu H.B., Wu X.J., Zhou Z.W., Chen G., Wang G.Q., Zhang L.Y., Li Y.F., Rajiv-Prasad K. (2009). The Prognostic Significance of Peripheral T-lymphocyte Subsets and Natural Killer Cells in Patients with Colorectal Cancer. Hepato-Gastroenterology.

[91] Baker K., Zlobec I., Tornillo L., Terracciano L., Jass J.R., Lugli A. (2007). Differential significance of tumour infiltrating lymphocytes in sporadic mismatch repair deficient versus proficient colorectal cancers: A potential role for dysregulation of the transforming growth factor-beta pathway. Eur. J. Cancer.

[92] McAllister F., Housseau F., Sears C.L. (2014). Microbiota and Immune Responses in Colon Cancer More to Learn. Cancer J..

[93] de Waal G.M., de Villiers W.J.S., Forgan T., Roberts T., Pretorius E. (2020). Colorectal cancer is associated with increased circulating lipopolysaccharide, inflammation and hypercoagulability. Sci. Rep..

[94] Yu A.I., Zhao L., Eaton K.A., Ho S., Chen J., Poe S., Becker J., Gonzalez A., McKinstry D., Hasso M. (2020). Gut Microbiota Modulate CD8 T Cell Responses to Influence Colitis-Associated Tumorigenesis. Cell Rep..

[95] Montalban-Arques A., Scharl M. (2019). Intestinal microbiota and colorectal carcinoma: Implications for pathogenesis, diagnosis, and therapy. EBioMedicine.

[96] Li S., Konstantinov S.R., Smits R., Peppelenbosch M.P. (2017). Bacterial Biofilms in Colorectal Cancer Initiation and Progression. Trends Mol. Med..

[97] Tetz G.V., Ruggles K.V., Zhou H., Heguy A., Tsirigos A., Tetz V. (2017). Bacteriophages as potential new mammalian pathogens. Sci. Rep..

[98] Chang K., Taggart M.W., Reyes-Uribe L., Borras E., Riquelme E., Barnett R.M., Leoni G., San Lucas F.A., Catanese M.T., Mori F. (2018). Immune Profiling of Premalignant Lesions in Patients With Lynch Syndrome. JAMA Oncol..

[99] Hegazy A.N., West N.R., Stubbington M.J.T., Wendt E., Suijker K.I.M., Datsi A., This S., Danne C., Campion S., Duncan S.H. (2017). Circulating and Tissue-Resident CD4^(+)^ T Cells With Reactivity to Intestinal Microbiota Are Abundant in Healthy Individuals and Function Is Altered During Inflammation. Gastroenterology.

[100] Sorini C., Cardoso R.F., Gagliani N., Villablanca E.J. (2018). Commensal Bacteria-Specific CD4^(+)^ T Cell Responses in Health and Disease. Front. Immunol..

[101] Maslowski K.M., Vieira A.T., Ng A., Kranich J., Sierro F., Yu D., Schilter H.C., Rolph M.S., Mackay F., Artis D. (2009). Regulation of inflammatory responses by gut microbiota and chemoattractant receptor GPR43. Nature.

[102] Geuking M.B., Cahenzli J., Lawson M.A.E., Ng D.C.K., Slack E., Hapfelmeier S., McCoy K.D., Macpherson A.J. (2011). Intestinal Bacterial Colonization Induces Mutualistic Regulatory T Cell Responses. Immunity.

[103] Sampson T.R., Debelius J.W., Thron T., Janssen S., Shastri G.G., Ilhan Z.E., Challis C., Schretter C.E., Rocha S., Gradinaru V. (2016). Gut Microbiota Regulate Motor Deficits and Neuroinflammation in a Model of Parkinson’s Disease. Cell.

[104] Feng T., Wang L.F., Schoeb T.R., Elson C.O., Cong Y.Z. (2010). Microbiota innate stimulation is a prerequisite for T cell spontaneous proliferation and induction of experimental colitis. J. Exp. Med..

[105] Lodes M.J., Cong Y.Z., Elson C.O., Mohamath R., Landers C.J., Targan S.R., Fort M., Hershberg R.M. (2004). Bacterial flagellin is a dominant antigen in Crohn disease. J. Clin. Investig..

[106] Chung H.C., Pamp S.J., Hill J.A., Surana N.K., Edelman S.M., Troy E.B., Reading N.C., Villablanca E.J., Wang S., Mora J.R. (2012). Gut Immune Maturation Depends on Colonization with a Host-Specific Microbiota. Cell.

[107] Yang J., Li D.F., Yang Z.Y., Dai W.K., Feng X., Liu Y.H., Jiang Y.Q., Li P.G., Li Y.H., Tang B. (2020). Establishing high-accuracy biomarkers for colorectal cancer by comparing fecal microbiomes in patients with healthy families. Gut Microbes.

[108] Yachida S., Mizutani S., Shiroma H., Shiba S., Nakajima T., Sakamoto T., Watanabe H., Masuda K., Nishimoto Y., Kubo M. (2019). Metagenomic and metabolomic analyses reveal distinct stage-specific phenotypes of the gut microbiota in colorectal cancer. Nat. Med..

[109] Nakatsu G., Li X.C., Zhou H.K., Sheng J.Q., Wong S.H., Wu W.K.K., Ng S.C., Tsoi H., Dong Y.J., Zhang N. (2015). Gut mucosal microbiome across stages of colorectal carcinogenesis. Nat. Commun..

[110] Mori G., Rampelli S., Orena B.S., Rengucci C., De Maio G., Barbieri G., Passardi A., Gardini A.C., Frassineti G.L., Gaiarsa S. (2018). Shifts of Faecal Microbiota During Sporadic Colorectal Carcinogenesis. Sci. Rep..

[111] Feng Q., Liang S.S., Jia H.J., Stadlmayr A., Tang L.Q., Lan Z., Zhang D.Y., Xia H.H., Xu X.Y., Jie Z.Y. (2015). Gut microbiome development along the colorectal adenoma-carcinoma sequence. Nat. Commun..

[112] Baxter N.T., Ruffin M.T., Rogers M.A.M., Schloss P.D. (2016). Microbiota-based model improves the sensitivity of fecal immunochemical test for detecting colonic lesions. Genome Med..

[113] Flemer B., Lynch D.B., Brown J.M.R., Jeffery I.B., Ryan F.J., Claesson M.J., O’Riordain M., Shanahan F., O’Toole P.W. (2017). Tumour-associated and non-tumour-associated microbiota in colorectal cancer. Gut.

[114] Wirbel J., Pyl P.T., Kartal E., Zych K., Kashani A., Milanese A., Fleck J.S., Voigt A.Y., Palleja A., Ponnudurai R. (2019). Meta-analysis of fecal metagenomes reveals global microbial signatures that are specific for colorectal cancer. Nat. Med..

[115] Thomas A.M., Manghi P., Asnicar F., Pasolli E., Armanini F., Zolfo M., Beghini F., Manara S., Karcher N., Pozzi C. (2019). Metagenomic analysis of colorectal cancer datasets identifies cross-cohort microbial diagnostic signatures and a link with choline degradation. Nat. Med..

[116] Abdulamir A.S., Hafidh R.R., Bakar F.A. (2011). The association of *Streptococcus bovis/gallolyticus* with colorectal tumors: The nature and the underlying mechanisms of its etiological role. J. Exp. Clin. Cancer Res..

[117] Vecherkovskaya M.F., Tetz G.V., Tetz V.V. (2014). Complete genome sequence of the *Streptococcus* sp. strain VT 162, isolated from the saliva of pediatric oncohematology patients. Genome Announc..

[118] Lu X.-J., Kang Q., Jin P., Sheng J. (2017). The Interactions Between Gut Microbiota and Lynch Syndrome. Clin. Gastroenterol. Hepatol..

[119] Yan Y., Drew D.A., Markowitz A., Lloyd-Price J., Abu-Ali G., Nguyen L.H., Tran C., Chung D.C., Gilpin K.K., Meixell D. (2020). Structure of the Mucosal and Stool Microbiome in Lynch Syndrome. Cell Host Microbe.

[120] Mori G., Orena B.S., Cultrera I., Barbieri G., Albertini A.M., Ranzani G.N., Carnevali I., Tibiletti M.G., Pasca M.R. (2019). Gut Microbiota Analysis in Postoperative Lynch Syndrome Patients. Front. Microbiol..

[121] Kostic A.D., Chun E.Y., Robertson L., Glickman J.N., Gallini C.A., Michaud M., Clancy T.E., Chung D.C., Lochhead P., Hold G.L. (2013). *Fusobacterium nucleatum* Potentiates Intestinal Tumorigenesis and Modulates the Tumor-Immune Microenvironment. Cell Host Microbe.

[122] Ferrarese R., Zuppardo R.A., Mannucci A., Antoci G., Ditonno I., Notaristefano C., Patricelli M.G., Raucci A.R., Di Leo M., Testoni P.A. (2019). Preliminary Data on Oral and Fecal Microbiota in Patients Affected by Lynch Syndrome. Gastroenterology.

[123] Dejea C.M., Wick E.C., Hechenbleikner E.M., White J.R., Welch J.L.M., Rossetti B.J., Peterson S.N., Snesrud E.C., Borisy G.G., Lazarev M. (2014). Microbiota organization is a distinct feature of proximal colorectal cancers. Proc. Natl. Acad. Sci. USA.

[124] Johnson C.H., Dejea C.M., Edler D., Hoang L.T., Santidrian A.F., Felding B.H., Ivanisevic J., Cho K., Wick E.C., Hechenbleikner E.M. (2015). Metabolism Links Bacterial Biofilms and Colon Carcinogenesis. Cell Metab..

[125] Swidsinski A., Loening-Baucke V., Theissig F., Engelhardt H., Bengmark S., Koch S., Lochs H., Dorffel Y. (2007). Comparative study of the intestinal mucus barrier in normal and inflamed colon. Gut.

[126] Leavitt J., Saleh N. (2019). The Microbiome and Colorectal Cancer: Current Clinical Trials. Oncology.

[127] Clinicaltrials.gov. https://clinicaltrials.gov/.

[128] Amin M.B., Greene F.L., Edge S.B., Compton C.C., Gershenwald J.E., Brookland R.K., Meyer L., Gress D.M., Byrd D.R., Winchester D.P. (2017). The Eighth Edition AJCC Cancer Staging Manual: Continuing to build a bridge from a population-based to a more “personalized” approach to cancer staging. CA Cancer J. Clin..

[129] Prorok-Hamon M., Friswell M.K., Alswied A., Roberts C.L., Song F., Flanagan P.K., Knight P., Codling C., Marchesi J.R., Winstanley C. (2014). Colonic mucosa-associated diffusely adherent afaC plus *Escherichia coli* expressing *lpfA* and *pks* are increased in inflammatory bowel disease and colon cancer. Gut.

[130] Conche C., Greten F.R. (2018). Fungi Enter the Stage of Colon Carcinogenesis. Immunity.

[131] Malik A., Sharma D., Malireddi R.K.S., Guy C.S., Chang T.C., Olsen S.R., Neale G., Vogel P., Kanneganti T.D. (2018). SYK-CARD9 Signaling Axis Promotes Gut Fungi-Mediated Inflammasome Activation to Restrict Colitis and Colon Cancer. Immunity.

[132] Wang T.T., Fan C.G., Yao A.R., Xu X.W., Zheng G.X., You Y., Jiang C.Y., Zhao X.Q., Hou Y.Y., Hung M.C. (2018). The Adaptor Protein CARD9 Protects against Colon Cancer by Restricting Mycobiota-Mediated Expansion of Myeloid-Derived Suppressor Cells. Immunity.

[133] Luan C.G., Xie L.L., Yang X., Miao H.F., Lv N., Zhang R.F., Xiao X., Hu Y.F., Liu Y.L., Wu N. (2015). Dysbiosis of Fungal Microbiota in the Intestinal Mucosa of Patients with Colorectal Adenomas. Sci. Rep..

[134] Coker O.O., Nakatsu G., Dai R.Z., Wu W.K.K., Wong S.H., Ng S.C., Chan F.K.L., Sung J.J.Y., Yu J. (2019). Enteric fungal microbiota dysbiosis and ecological alterations in colorectal cancer. Gut.

[135] Wirth F., Goldani L.Z. (2012). Epidemiology of *Rhodotorula*: An emerging pathogen. Interdiscip. Perspect. Infect. Dis..

[136] Gaitanis G., Magiatis P., Hantschke M., Bassukas I.D., Velegraki A. (2012). The *Malassezia* Genus in Skin and Systemic Diseases. Clin. Microbiol. Rev..

[137] Shkoporov A.N., Clooney A.G., Sutton T.D.S., Ryan F.J., Daly K.M., Nolan J.A., McDonnell S.A., Khokhlova E.V., Draper L.A., Forde A. (2019). The Human Gut Virome Is Highly Diverse, Stable, and Individual Specific. Cell Host Microbe.

[138] Dahiya D.K., Renuka (2017). The gut virome: A neglected actor in colon cancer pathogenesis. Future Microbiol..

[139] Hannigan G.D., Duhaime M.B., Ruffin M.T.T., Koumpouras C.C., Schloss P.D. (2018). Diagnostic Potential and Interactive Dynamics of the Colorectal Cancer Virome. mBio.

[140] Burns M.B., Montassier E., Abrahante J., Priya S., Niccum D.E., Khoruts A., Starr T.K., Knights D., Blekhman R. (2018). Colorectal cancer mutational profiles correlate with defined microbial communities in the tumor microenvironment. PLoS Genet..

[141] Dale H.F., Rasmussen S.H., Asiller O.O., Lied G.A. (2019). Probiotics in Irritable Bowel Syndrome: An Up-to-Date Systematic Review. Nutrients.

[142] Fong W.N., Li Q., Yu J. (2020). Gut microbiota modulation: A novel strategy for prevention and treatment of colorectal cancer. Oncogene.

[143] Boyle R.J., Robins-Browne R.M., Tang M.L.K. (2006). Probiotic use in clinical practice: What are the risks?. Am. J. Clin. Nutr..

[144] Cheng W.Y., Wu C.Y., Yu J. (2020). The role of gut microbiota in cancer treatment: Friend or foe?. Gut.

[145] Yuan L.S.R., Zhang S., Li H., Yang F., Mushtaq N., Ullah S., Shi Y., An C., Xu J. (2018). The influence of gut microbiota dysbiosis to the efficacy of 5-Fluorouracil treatment on colorectal cancer. Biomed. Pharm..

[146] Osterlund P., Ruotsalainen T., Korpela R., Saxelin M., Ollus A., Valta P., Kouri M., Elomaa I., Joensuu H. (2007). *Lactobacillus* supplementation for diarrhoea related to chemotherapy of colorectal cancer: A randomised study. Brit. J. Cancer.

[147] Zaharuddin L., Mokhtar N.M., Nawawi K.N.M., Ali R.A.R. (2019). A randomized double-blind placebo-controlled trial of probiotics in post-surgical colorectal cancer. BMC Gastroenterol..

[148] Lamichhane P., Maiolini M., Alnafoosi O., Hussein S., Alnafoosi H., Umbela S., Richardson T., Alla N., Lamichhane N., Subhadra B. (2020). Colorectal Cancer and Probiotics: Are Bugs Really Drugs?. Cancers.

[149] Agraib L.M.A.-S.A., Salah S., Abu-hijlih R., Abuhijla F. (2020). The effect of probiotics supplementation on the side effects of chemo radiotherapy for colorectal cancer: A literature review. Oncol. Radiother..

[150] Chen D.F., Jin D.C., Huang S.M., Wu J.Y., Xu M.Q., Liu T.Y., Dong W.X., Liu X., Wang S.A., Zhong W.L. (2020). *Clostridium butyricum*, a butyrate-producing probiotic, inhibits intestinal tumor development through modulating Wnt signaling and gut microbiota. Cancer Lett..

[151] Cho J.R., Yoon B.J., Oh H.K. (2019). Effect of Probiotics on Bowel Function Restoration after Ileostomy Reversal in Patients with Rectal Cancer: A Double-Blind Randomized Controlled Trial. Gastroenterology.

[152] Friederich P., Verschuur J., van Heumen B.W.H., Roelofs H.M.J., Berkhout M., Nagtegaal I.D., van Oijen M.G.H., van Krieken J.H.J.M., Peters W.H.M., Nagengast F.M. (2011). Effects of intervention with sulindac and inulin/VSL#3 on mucosal and luminal factors in the pouch of patients with familial adenomatous polyposis. Int. J. Colorectal Dis..

[153] Sivan A., Corrales L., Hubert N., Williams J.B., Aquino-Michaels K., Earley Z.M., Benyamin F.W., Lei Y.M., Jabri B., Alegre M.L. (2015). Commensal *Bifidobacterium* promotes antitumor immunity and facilitates anti-PD-L1 efficacy. Science.

[154] Vetizou M., Pitt J.M., Daillere R., Lepage P., Waldschmitt N., Flament C., Rusakiewicz S., Routy B., Roberti M.P., Duong C.P. (2015). Anticancer immunotherapy by CTLA-4 blockade relies on the gut microbiota. Science.

[155] Iida N., Dzutsev A., Stewart C.A., Smith L., Bouladoux N., Weingarten R.A., Molina D.A., Salcedo R., Back T., Cramer S. (2013). Commensal bacteria control cancer response to therapy by modulating the tumor microenvironment. Science.

[156] Mager L.F., Burkhard R., Pett N., Cooke N.C.A., Brown K., Ramay H., Paik S., Stagg J., Groves R.A., Gallo M. (2020). Microbiome-derived inosine modulates response to checkpoint inhibitor immunotherapy. Science.

[157] Turroni F., Foroni E., Pizzetti P., Giubellini V., Ribbera A., Merusi P., Cagnasso P., Bizzarri B., de’Angelis G.L., Shanahan F. (2009). Exploring the Diversity of the Bifidobacterial Population in the Human Intestinal Tract. Appl. Environ. Microb..

[158] Bardhan K., Anagnostou T., Boussiotis V.A. (2016). The PD1:PD-L1/2 Pathway from Discovery to Clinical implementation. Front. Immunol..

[159] Ohaegbulam K.C., Assal A., Lazar-Molnar E., Yao Y., Zang X.X. (2015). Human cancer immunotherapy with antibodies to the PD-1 and PD-L1 pathway. Trends Mol. Med..

[160] Sears C.L., Pardoll D.M. (2018). The intestinal microbiome influences checkpoint blockade. Nat. Med..

[161] Shui L., Yang X., Li J., Yi C., Sun Q., Zhu H. (2020). Gut Microbiome as a Potential Factor for Modulating Resistance to Cancer Immunotherapy. Front. Immunol..

[162] Timmermann B., Kerick M., Roehr C., Fischer A., Isau M., Boerno S.T., Wunderlich A., Barmeyer C., Seemann P., Koenig J. (2010). Somatic Mutation Profiles of MSI and MSS Colorectal Cancer Identified by Whole Exome Next Generation Sequencing and Bioinformatics Analysis. PLoS ONE.

[163] Humphries A., Daud A. (2018). The gut microbiota and immune checkpoint inhibitors. Hum. Vaccines Immunother..

[164] Temraz S., Nassar F., Nasr R., Charafeddine M., Mukherji D., Shamseddine A. (2019). Gut Microbiome: A Promising Biomarker for Immunotherapy in Colorectal Cancer. Int. J. Mol. Sci..

[165] Ben Q.W., Sun Y.W., Chai R., Qian A.H., Xu B., Yuan Y.Z. (2014). Dietary Fiber Intake Reduces Risk for Colorectal Adenoma: A Meta-analysis. Gastroenterology.

[166] Tanoue T., Morita S., Plichta D.R., Skelly A.N., Suda W., Sugiura Y., Narushima S., Vlamakis H., Motoo I., Sugita K. (2019). A defined commensal consortium elicits CD8 T cells and anti-cancer immunity. Nature.

[167] Zackular J.P., Baxter N.T., Chen G.Y., Schloss P.D. (2015). Manipulation of the Gut Microbiota Reveals Role in Colon Tumorigenesis. mSphere.

[168] Bullman S., Pedamallu C.S., Sicinska E., Claney T.E., Zhang X.Y., Cai D.N., Neuberg D., Huang K., Guevara F., Nelson T. (2017). Analysis of *Fusobacterium* persistence and antibiotic response in colorectal cancer. Science.

[169] Zhao S., Gao G.H., Li W., Li X.F., Zhao C., Jiang T., Jia Y.J., He Y.Y., Li A.W., Su C.X. (2019). Antibiotics are associated with attenuated efficacy of anti-PD-1/PD-L1 therapies in Chinese patients with advanced non-small cell lung cancer. Lung Cancer.

[170] Dong X., Pan P., Zheng D.W., Bao P., Zeng X., Zhang X.Z. (2020). Bioinorganic hybrid bacteriophage for modulation of intestinal microbiota to remodel tumor-immune microenvironment against colorectal cancer. Sci. Adv..

[171] Moayyedi P., Yuan Y.H., Baharith H., Ford A.C. (2017). Faecal microbiota transplantation for *Clostridium difficile*-associated diarrhoea: A systematic review of randomised controlled trials. Med. J. Aust..

[172] Chang C.W., Lee H.C., Li L.H., Chiau J.S.C., Wang T.E., Chuang W.H., Chen M.J., Wang H.Y., Shih S.C., Liu C.Y. (2020). Fecal Microbiota Transplantation Prevents Intestinal Injury, Upregulation of Toll-Like Receptors, and 5-Fluorouracil/Oxaliplatin-Induced Toxicity in Colorectal Cancer. Int. J. Mol. Sci..

[173] Wang Q., Ye J.Z., Fang D.Q., Lv L.X., Wu W.R., Shi D., Li Y.T., Yang L.Y., Bian X.Y., Wu J.J. (2020). Multi-omic profiling reveals associations between the gut mucosal microbiome, the metabolome, and host DNA methylation associated gene expression in patients with colorectal cancer. BMC Microbiol..

[174] Wilson M.R., Jiang Y.D., Villalta P.W., Stornetta A., Boudreau P.D., Carra A., Brennan C.A., Chun E., Ngo L., Samson L.D. (2019). The human gut bacterial genotoxin colibactin alkylates DNA. Science.

